# Pathology and cancer in Africa

**DOI:** 10.3332/ecancer.2019.945

**Published:** 2019-07-25

**Authors:** Kenneth Fleming

**Affiliations:** Green Templeton College, University of Oxford, 43 Woodstock Road, Oxford OX2 6HG, UK

**Keywords:** cancer, Africa, low access to pathology

## Abstract

In high-income countries, it would be inconceivable to treat a tumour when its pathology is unknown. However, this can be the case among patients in sub-Saharan Africa. The absence of pathologists and the resultant delays in reporting contribute to patients being treated before the nature of the lesion is known. This is compounded by the frequent absence of auxiliary tests to better define tumour characteristics.

## Introduction

Pathology is crucial for the diagnosis and management of any cancer. This applies to all branches of pathology. For example, measurement, in clinical chemistry labs, of blood levels of molecules, such as CEA (carcino-embryonic antigen) and PSA (prostate specific antigen), is important in assessing the response to therapy of patients with colorectal and prostate cancer. Microbiology is vital in managing infections in immune-suppressed cancer patients, while blood transfusion is necessary in many patients with haematological and other cancers. Moreover, much of the workload of the haematologist is related to diagnosis and management of haematological malignancies and recently, with the rise of immune modulation for cancer therapy, immunologists have become more deeply involved in cancer care. As cell, or anatomic, pathology is most closely identified with diagnosis and management of cancer, this short chapter will focus on the situation of cell/anatomic pathology (hereafter, pathology) in sub Saharan Africa.

## Current situation

Hard data are few and far between, but there are two main issues: insufficient access and variable standards. Poor access results from a complex of problems: insufficient workforce, poor infrastructure, relative absence of integrated networks of laboratories and inadequate financing. Variable standards reflect inadequate training, lack of specialised tests like immunohistochemistry and patchy use of quality assurance schemes and accreditation systems.

## Insufficient access

The WHO 2015 Non-communicable Disease Country Capacity Survey found that only 35% of low-income countries reported having anatomic pathology services generally available in the public health sector—where ‘generally available’ is defined as being accessible in 50% or more of health care facilities within the country [1]. The situation in Africa reflects this. Pathology services are predominantly concentrated in larger towns and cities, with limited or no provision in rural communities. For example, the 2018 IARC publication on Cancer in Sub-Saharan Africa showed wide variation in rates of tissue diagnosis by both location of the registry and cancer site [2]. In general, large cities had rates of around 80% for most tumours, but even some common tumour types had low rates—e.g., 45% for colorectal cancer in males in Kampala. In poorer countries, rates were much lower—e.g., in Niamey, Niger, the rate for all sites was around 40%. It is worth noting that the registries obtained their data mainly from hospitals and pathology departments and did not include the patients who did not attend either. Thus, although the proportion of cancer patients who do not have a tissue diagnosis before treatment is unknown, if one includes patients who do not present to hospital, it is likely to be considerably lower than in high-income countries.

### Pathologist numbers

While there are several reasons for the above, a major problem is the lack of a trained workforce, especially pathologists. Unlike some other branches of pathology, cell/anatomic pathology is almost completely dependent on trained pathologists. A recent paper on the number of pathologists in Africa, using a snowball/convenience approach and a questionnaire sent to a number of individual pathologists and organisations, showed the distribution of pathologists in Africa [3]. The three main messages were first, the average number of pathologists per head of population is 1/1,000,000. This compares with the ratio of 1 pathologist to 15–20,000 in the US and UK, a difference of between 50- and 70-fold. Second, there are three main concentrations—South Africa, West Africa—Nigeria/Ghana—and East Africa, especially Kenya, Ethiopia Uganda and Tanzania. Outside these areas, numbers become very low. Third, only seven countries have a ratio of less than 1/1,000,000. At least one country has no pathologist.

Related to this, there is insufficient educational capacity. At present, it appears that in sub-Saharan Africa (SSA), about 80 pathologists/year graduate [4]. Taking into account, a probable wastage rate of around 50%–10% (retirements etc.), it would take around 400 years for SSA to reach the US/UK ratio of pathologists/population. This does not take into account the projected increase in population of the continent, which is predicted to double by 2050. Whether attaining a similar ratio of pathologists/population as in the UK/USA is an appropriate target can be debated, but clearly there is a current and future severe shortage of trained pathologists.

### Other factors

As mentioned above, several other factors contribute to the poor access to pathology. Infrastructure (electricity, water, space, equipment, reagents and IT) is weak throughout SSA, with, for example, not infrequent power and stock outages. Maintenance of equipment is minimal, largely because of the unaffordability of maintenance contracts. Very few countries have integrated tiered networks of labs, a structure which can help to share workforce and training and maximise limited resources in general. Underlying all of the above is inadequate financing. A recent paper [5] estimated that fewer than 6% of countries had substantive discussion about financing of the laboratory systems in their National Strategic Laboratory Plan (only 40% of countries had such a Plan). The same paper recommended that the share of health spend on laboratories (all pathology disciplines) should be about 5%—in South Africa it was 3.5%, while that in Uganda ranged from 3.3%–4.6%. It is likely that most other countries spend considerably less. A related factor is that in many countries, the user pays. This of course biases against the poorer patient, which predominately includes the rural population, and contributes to insufficient access.

## Standards

Even when tissue diagnosis is available, there are concerns about the quality of the reports. Although hard data are rare, several papers have examined some of the issues. A retrospective assessment of reports of breast carcinoma in the university teaching hospital in Lagos, Nigeria in 2016, showed that 60% of the reports did not mention lymph node status and 50% did not mention excision margins [6]. Hormone receptor was not stated in 74% cases. More concerning is that when the cases were examined by pathologists in the UK, there was a 46.9% discordance rate in the basic diagnosis, with failure to record the type of breast cancer in 72.2% reports. A paper on Turn Around Time (TAT)—time from biopsy till receipt of report by clinician—from Malawi [7] showed that the median TAT for unpaid samples was 71 days, obviously making the report of limited or no value in patient diagnosis and treatment.

Given the lack of reliable data from many countries, it is unclear how representative the above data are. Undoubtedly there are laboratories, especially private ones, where standards are high, but, anecdotally, standards in the majority of laboratories are more similar to the above than not.

At a general level, addressing the many issues around standards is probably best achieved through participation in some form of quality assurance system (both internal and external), usually, but not necessarily, as part of an accreditation programme. Apart from South Africa, unfortunately relatively few laboratories participate in such programmes. While most of the labs involved were not cell pathology and were private, an analysis of the over 900 laboratories in Kampala in 2013, showed that only 5% reached the minimal level of the SLIPTA system of accreditation [8].

At a local level, specific initiatives can make substantial improvements. In Malawi, with external help from the University of North Carolina and recruitment of pathologists, the TAT has been reduced to 5 days for paid samples and to between 10 and 40 days for unpaid samples (compared to the previous 70 days) [9]. In Rwanda, when the cancer centre was set up in Butaro in 2012, where slides were prepared and sent to Boston, the median TAT was 30 days. Static image telepathology reduced that to 14 days, but the use of visiting pathologists reduced this to 5 days [10]. A clear lesson from both these examples is the major benefits of having on-site pathologists.

## Conclusion

The combination of insufficient access to, and the variable standards of pathology in sub-Saharan Africa outlined above, undoubtedly means that a not insignificant proportion of cancer patients are receiving untimely and/or inaccurate diagnosis. This has important repercussions for the patients and their families, not least in unnecessary prolongation of illness, or even unnecessary death. However, it also has significant economic impact with scarce resources being used for the wrong diagnosis, and significant economic output being lost because patients are off work unnecessarily. Recognition of this problem is increasing and specific solutions are being initiated.

## Conflicts of interest

The author has no conflicts of interest to report.

## Funding

Senior Adviser for Pathology, Center for Global Health, NCI, Washington, 2015-18 (part-time external contractor).
